# An Essay on Measles in the Adult

**Published:** 1847-10-01

**Authors:** 


Memoire sur la Rougeole des Adultes. Par le Docteur *Michel Lévy.*

An Essay on Measles in the Adult. By *M. Levy*, M.D.; Chief Physician and Senior Professor of the Hospital of Instruction at Metz; late Professor at the Val-de-Grâce, &c. Octavo, pp. 48. Paris, 1847.

This *brochure* from the pen of Professor Levy, details the particulars of an epidemic of Measles in the Adult, which he has recently had the opportunity of witnessing at the Clinical Hospital at Metz, over which he so ably presides. The conclusions he has arrived at upon several interesting circumstances connected with the disease are well worthy the notice of our readers.

"The measles," he observes, "though manifesting itself less frequently among adults than children, is far from sparing these former to the extent supposed by several pathologists who have drawn the elements of their statistics exclusively from hospitals. In the most recent analytical work on medicine, the Compendium of MM. Monneret and Fleury, we find the statement—'After fifteen years the measles become rare.' This does not agree with the clinical observations at the military hospitals, which receive a large proportion of patients between the ages of 18 and 30. In these we are constantly meeting with cases of measles. In the east, south, and north of France, in Corsica, and the Morea, wherever, indeed, the vicissitudes of our career have taken us, we have seen young soldiers seized with measles, and frequently the affection attains in our garrisons the magnitude of an epidemic. During the siege of Antwerp, the ambulance at Boom, the surgical service of which was under my direction, received a certain number of cases of measles; and ten years' experience at Val-de-Grâce enabled me to observe these cases occurring periodically, and almost always proportionally to the extent the disease prevailed in the town. In 1837, there were 60 cases alone in the service under my charge, the disease prevailing at the same time epidemically at Paris, Versailles, and other places. The foundation of the present work is constituted by the facts I have observed in the hospital at Metz during thirteen months, from the 1st Jan. 1846 to 28th Feb. 1847. It is first necessary to make the remark that the distinction which is judiciously drawn between the diseases of civil life and those of garrisons, is not applicable in respect to our subject. During the period embraced in our observation, the progress of measles has been precisely the same in these two groups of the population. Absent or sporadic in the town it has been equally wanting in the hospital, or was only seen there now and then: the attacks becoming multiplied in the city, it soon became epidemic in the garrison. The parallel was constant."

The number of cases observed during these 13 months amounted to 120, three patients only were less than 18 years of age, the oldest being 34.

*Form of the Eruption.*—Generally this was confluent, the face being almost always vultuous. In 10 cases the confluence was excessive. The papular form (boutonneuse) was observed in four cases. In four others the eruption was anormal, being scarcely observable on the body, while the limbs were covered with large numbers of bright red spots. Two patients had a violaceous suffusion of the face, and a discrete eruption over the body.

*Progress of the Eruption.*—In 99 out of the 120 cases this went through its regular stages. Delitescence took place in 21—10 times on the second, and 16 times on the third day. In 7 cases cold seemed to be the cause of the sudden disappearance of the eruption, and in 3 the delitescence coincided with intense diarrhœa; but diarrhœa manifested itself in many other cases without deranging the course of the eruption. As to the consequences of the suppression of the eruption there were none remarked in 14 patients, who were as promptly cured as if the eruption had pursued its usual course. Two were seized with diarrhœa and one with vomiting, but soon recovered. In another, varicella supervened, and in another, signs of phthisis. In six, severe bronchitis followed; and two died subsequently to the retrocession, one having succumbed to an assemblage of thoracic complications, and the other to severe capillary bronchitis and acute dysentery supervening upon the administration of tartar-emetic. Of these pulmonary complications M. Levy thus speaks:

"Dyspnœa, oppression, intense cough, bright coloration of the face, and even anxiety, are not certain indications of an inflammatory complication of the small bronchi or the pulmonary parenchyma. We have seen these symptoms carried to a high degree in measles with slight catarrh as fugacious as the exanthem; and then they disappeared as if by enchantment when the eruption had entirely come out. They are only the expression of a general labouring of the organism, resolving itself in the eruption. When they subsist after the complete evolution of this, they announce the co-existence, not of an ordinary bronchitis, but of a suffocative catarrh (capillary bronchitis), or of an inflammation (almost always lobular) of the lung itself."

*Concomitants.*—(1.) As a precursory symptom *Epistaxis* was of fequent occurrence, and in 11 patients appeared during the course of the eruption, and generally on several occasions. (2.) *Vomiting*, which is seldomer a precursory symptom than in small-pox, was observed in seven after the appearance of the rash. (3.) The precursory *Diarrhœa* did not derange the course of the eruption in any of the cases in which it appeared, and probably may be considered as a mere effect of the morbillous hyperæmia of the intestinal mucous membrane, just as weeping is of the ocular, or coryza of the pituitary. It is to be distinguished from that which is induced by a real complication, as from enteritis, &c., from which it may be distinguished by its coincidence with the other precursory symptoms, its mucous character, the comparative infrequency of the stools—these seldom exceeding 6 or 8 in the 24 hours—the absence of abdominal pain, and its non-perturbatory power over the cutaneous eruption. In 30 patients this diarrhœa appeared. It was not interfered with, and it is to be remarked that the subjects of it suffered less from the morbillous bronchitis. (4.) *Constipation* was observed only in three patients. (5.) With four exceptions, *all* the patients offered the symptoms of *bronchial catarrh.*

"These symptoms are those of *bronchitis*, but is the modification which measles impresses upon the air-passages identical with phlegmasia of this membrane? If it is true that in this pyrexia the amount of fibrine continues stationary, or even in severe cases falls below the normal mean, we may, from this alone, conclude that the bronchial lesion, which nineteen out of twenty times accompanies measles, is not inflammatory, or at all events cannot be classed with the pure phlegmasiæ of the respiratory organs. The above-named symptoms do not absolutely characterize inflammation of the bronchi; for we observe them whenever these become the seat of active or passive congestion, as after the first week of typhoid fever, and in the advanced lesions of the heart. A simple hyperæmia of the bronchial mucous membrane, analogous to that which, directed on the reticular texture of the skin, determines the production of the spots of measles, may give rise to symptoms of bronchitis. Later, in a certain number of cases, inflammation does supervene, but it participates in the special nature of the congestion which has prepared the way for it. In this way we explain the rubeolic catarrh, to which we may add coryza, angina, ophthalmia, and diarrhœa.

"If the patient is surrounded by a temperature and the care his case requires, this follows the phases of the exanthem and does not survive it. If, however, he is imprudently exposed to the cold, the severity of the weather renders it difficult to protect him from this, or if he is predisposed to diseases of the respiratory organs, then it is by no means uncommon to find this morbillous hyperæmia of the bronchi pass into an inflammatory type, and retard convalescence, however regular the course of the eruption may have been. The measly eruption, too, may become propagated throughout the whole extent of the bronchial tree, and from the very commencement of the rubeola symptoms of a deep-seated bronchitis may coincide with those of the exanthem. Nine of our patients offered striking examples of this. The case now becomes serious. The frequency of the circulation increases in an inverse degree to the amount of respiratory surface which remains disposable in the lungs. Wherever it is invaded by the exanthem, the bronchial surface re-acts less vigorously on the air, or may become totally unfit for this function. The more narrow the field for hæmatosis is rendered the more must the blood be hastened in its course to bring it a greater number of times in contact with the air. Hence violaceous colour of the face, especially of the mucous orifices; rapidity of pulse (120 to 140), and of inspiratory movements (48 to 60); rhonchus audible at a distance; vibratory and sub-crepitant râles heard over the entire chest; obstinate, harsh, paroxysmal cough, followed by the laborious expectoration of small quantities of tenacious and viscous matters, or of a white, foamy froth; an ever-increasing anxiety; and at last delirium. This is the spectacle such patients present, and, without the promptest aid, they rapidly fall into a state of asphyxia."

*Complications.*—(1.) The co-occurrence of several simultaneous eruptions was only observed towards the end of the epidemic. Eight patients then furnished examples of this. In three, varicella was commingled with the rubeola. In one, rubeola, varioloid, and miliaria were observed simultaneously: and, in another, with variolous pustules on the face, there was a scarlatina suffusion on the trunk, and spots of measles on the neck and limbs. In two other cases the measles was complicated with urticaria, and in another a purpura, which required 23 days for its cure, appeared. (2.) The most common complications were affections of the chest, in the form of bronchi-pneumonia, of which six examples are cited, one of these proving fatal, in consequence of the patient's neglecting its treatment, the eruption pursuing its normal course. (3.) Diseases of the digestive organs have rarely offered complications. These complications have also been equally rare in the various instances of measles in the adult which have on former occasions come under M. Levy's notice; while MM. Rilliet and Barthez noted the occurrence of gastro-enteric phlegmasia in 46 out of 167 cases of measles occurring in children. He agrees with MM. Blache and Guersant, that this difference is rather due to the cachectic condition of the children, who formed the object of those researches, than to the mere effect of age—a condition resulting from a long residence within the walls of a hospital, and usually leading to an entero-colitis or pneumonia.

*Consecutive Diseases.*—"The question of diseases consecutive to measles is of a great importance in practice; but science is far from being in possession of a sufficiency of materials for its solution. The difficulties it gives rise to depend upon the complexity of the etiological elements. We are constantly exposed to the deduction from the simple succession of two facts of a relation of causality which does not exist between them. Has the so-called consecutive disease closely followed the measles? It may depend upon the constitution of the subject, and upon the influences which modified it prior to the attack. If an interval has elapsed between the one and the other, who can say whether the new affection, whatever relationship it may seem to have with the measles, is not due to causes which necessarily escape the physician? However this may be, we were well placed to follow the ulterior phases of the health of our measles patients. On the one hand our observations extend over a period of thirteen months, and on the other hand scarcely any of the patients we have treated for the disease have quitted the garrison, but remained under the eyes of our colleagues in the different regiments, whose attention having been drawn to them by the memoranda we had placed on their tickets of dismissal, they were subjected to an attentive superintendence, and, in the case of their diseases requiring their return to the hospital, they were again consigned to our clinical wards."

In this manner 16 patients who had been attacked with measles came again under the author's care, and of whose cases he presents an analysis. The results, to which we can alone refer, are that eight of these cases presented examples of non-tubercular and eight of tubercular affections; the former consisting of bronchitis, pneumonia, or pleurisy, or two of these combined.

"The most general fact which results from the tables is, that all the diseases supervening after or springing up with measles have fallen upon the respiratory organs. Must we hence conclude that this disease impresses on these a greater aptitude to phlegmasiæ? Clinical observation replies affirmatively. Bronchial hyperæmia is one of the determinations of rubeola upon the internal tegument. Inflammation of the bronchi frequently spreads even to their capillary divisions, and lobular pneumonia is added in a great number of cases. These are facts acquired by science, and which the observation of our predecessors had long since established, with less anatomical precision, and according to different etiological views, which however detract nothing from the proper signification of facts. '*Infantes,*' says Sydenham, in 1670, '*præsertim huic malo (spirandi difficultati et tussiculæ importuniùs urgenti) sunt obnoxii, quod morbillis jam facessentibus se ostendit, unde in peripneumoniam conjiciuntur, quæ plures jugulat, quam aut variolæ ipsæ, aut symptoma quodcumque ad eum spectans morbum.*'

"Is the tuberculization which we have noticed in one half these cases at all connected with this inflammatory habitude of the respiratory organs, with the progressive anæmia of the patients who have suffered from rubeola, and who do not become re-established, or with any specific and not yet defined influence belonging to this eruptive fever? These problems will never obtain a rigorous solution. It is impossible to say whether these eight examples possess only the value of a simple coincidence with measles, or whether this has played a more active and direct part in the production of the tubercle. If it were established that the frequency of bronchial and pulmonary inflammation suffices to lead to its secretion, what we have already said of the power of measles to excite inflammation would furnish a reply to the question. Tubercle shews itself sufficiently frequently in children who have had measles; for, according to both Rilliet and Barthez, there is about one example of this in every eleven cases. M. Andral believes that measles accelerates the softening of pre-existing tubercle, an opinion which the facts we have collected do not positively confirm. Without carrying this discussion further, as it can lead to no certain proof, by reason of the insufficiency of clinical observations, we cannot admit with M. Rufz, 'that there does not exist in science a more hazardous proposition than the pretended influence of measles in the development of tubercle.' So many judicious observers of all epochs (Hoffmann, Stoll, Frank, Rayer, Guersant, &c.) have seen phthisis succeed to rubeola, that the relation which they have believed themselves able to perceive between these two affections, though deprived of the sanction of a positive demonstration, cannot be altogether destitute of truth. The facts above reported incline us to this opinion. Whether in the case of adults or children, the practitioner should examine with the greatest solicitude the state of the respiratory organs, both during the course, and after the disappearance of the disease."

*Antagonism.*—Measles seems to exert a certain salutary action against some of the chronic diseases of the skin; obstinate eczema, impetigo, &c., disappearing after the induction of congestion of the cutaneous surface by the new disease. MM. Blache and Guersant have also seen certain neuroses, as chorea and pertussis, cured under the influence of measles. M. Levy mentions two or three facts, shewing the occasional effect of the disease in inducing a suppression of long-standing gonorrhœa.

*Mortality.*—Of the 120 patients six died; a mortality far less than that attendant upon the disease in infancy. According to MM. Rilliet and Barthez, observing the disease however in a very bad class of subjects, uncomplicated measles is cured in five out of six cases, while, in the complicated form, one-half the cases fall victims. But these statements cannot be generalized, for in 1837 M. Levy had 16 deaths in only 60 adults.

"Still, the general result of our observations leads us to recognize that the measles causes a smaller proportion of deaths among adults than among children; and this difference, which we are unable to express in figures, but in our opinion is certain, is explained by the following circumstances. A greater resistance of the organism: a less liability to lobular pneumonia: a greater facility of clinical examination, and certainty in treatment. For the rest, the rubeola of adults, like that of children, follows the course of each epidemic. The prognosis depends essentially on that unknown *quid divinum* which measures out to epidemics their proportion and sphere of energy. Meteorological conditions of themselves furnish no element for such appreciation. The serious epidemic we witnessed in 1837 coincided with a mild temperature, while in that at Metz, during December, January, and February, the thermometer was almost always between 0° C. and 10° C. It is important to distinguish the mortality as arising from the rubeola itself from that dependent upon the consequences of the affection. Not one of the deaths occurred during the eruption nor immediately after its delitescence. Between the period of admission and that of death a delay of from 12 to 38 days occurred. The danger proceeds not then from the exanthem itself, but from the complications and consequences. 'If,' says Stoll, (Aphorism 578) 'more die in the small-pox than from its consequences, more die from the consequences of the measles than from the disease itself, and perhaps as many in this as in the other.'"

*Treatment.*—This is much the same as in the measles of children. In the precursory stage, hygienic means alone are called for, and no attempt need be made to treat the diarrhœa, vomiting, &c., which occur during the evolution of the eruption. The character of the epidemic must however determine us much on this point. Retrocession is not so grave an affair as is sometimes believed, and does not necessarily call for special medication. On its occurrence, the most careful exploration of the condition of each organ must be repeatedly made, in order to detect and meet any complication that may interfere with the progress of the eruption; but, this not being detected, an intelligent expectation is the best and safest practice. Suppose a splanchnic congestion or internal inflammation has become developed, it will be better to combat this directly, than by any endeavour to excite a tegumentary revulsion by the various internal and external stimuli usually recommended. "Of all the exanthematous eruptions none so fugaceous as that of measles; and could we recall it to the surface, it is very doubtful whether the partial hyperæmia of the dermis, which constitutes it, would prove powerful enough to displace a phlegmasia which had fixed upon so vascular an organ as the lungs." The great difficulty is of course the treatment of the pulmonary complications. "It might seem that men of from 18 to 30 years of age would bear *bleeding* better than children. It is not so. The inflammation here is not of a simple and legitimate character, but more approaches congestion in its nature; and is frequently accompanied by nervous erethism or prostration of strength." Bleeding induces rapid anæmia; and, on comparing his observations made at different epochs, M. Levy finds that this is not dependent upon the character of different epidemics; but that it is one of the consequences of the general modification which this disease impresses on the organism. *Blisters* are of some use; but the means from which he has derived most benefit is the *tartar-emetic*, given in nauseating doses in capillary bronchitis and in contra-stimulant doses in secondary pneumonia. In the first of these cases he gives it only on alternate days, so as not to distress too much the digestive organs. In the second, if the subject is strong, he precedes it by a venesection, and follows it by a blister. In some patients, *Kermes mineral* is supported better than the antimony; but it is a much more uncertain medicine.

We have confined ourselves to giving an analysis of this interesting little Essay. As opportunities for observing the disease on a large scale in the adult are rare in the civil practice of this country, and as the experience of our military hospitals is not communicated to the medical public with the promptitude and regularity it ought to be, we believe an account of the opinions of so good an observer as Dr. Levy will prove acceptable to our readers.

